# Thermovision Analysis Changes of Human Hand Surface Temperature in Cold Pressor Test

**DOI:** 10.1155/2015/783642

**Published:** 2015-08-12

**Authors:** Agnieszka Chwałczyńska, Katarzyna Gruszka, Ireneusz Całkosiński, Krzysztof A. Sobiech

**Affiliations:** ^1^Department of Human Biology, Faculty of Physiotherapy, University School of Physical Education, al. I. J. Paderewskiego 35, 51-612 Wroclaw, Poland; ^2^Faculty of Health Science, Wroclaw Medical University, Wybrzeże L. Pasteura 1, 50-367 Wroclaw, Poland

## Abstract

The cold pressor test (CTP) as a diagnostic method of the circulatory system reactivity may be a basis for the qualification for thermal stimulation therapy. The aim of the work was a thermovisual assessment of the reaction to the Hines and Brown cold pressor test. A group of 30 healthy men in the age of 23.5 ± 0.8 years were examined. The average weight of the examinees was 78.4 ± 9.2 kg, their height 180.7 ± 5.9 cms, and BMI 23.9 ± 2.2 kg/m^2^. A thermovisual picture of a tested and not tested hand of all the subjects was taken before and after the cold pressor test. Under the influence of cold water the surface temperature of a tested hand has decreased in a statistically significant way by 8.3°C on average, which is 29% of the temperature before the test, whilst the temperature of an untested hand dropped by 0.67°C. The decreases of temperature were not even and there was a statistically significant difference between the dorsal and palmar side of the hand. The correlation between the changes of systolic blood pressure and the hand surface temperature before and after CTP was observed.

## 1. Introduction

The cold pressor test, in which the vascular reactivity to lowering body temperature by immersing in cold water has been examined, was described by Hines Jr. and Brown [1–4]. The action of the stimulus, which in case of CTP is icy cold water, results in a double phase activation of thermal receptors by blood vessels stricture in the first phase and the enhancement of blood flow in the second phase [5]. Internal reaction of the cardiovascular system is noticed externally in the first short phase as skin pallor, so called “goosebumps” and a temporary feeling of cold. In the second phase, the blood vessels congestion leads to a vascular reaction noticeable in strong skin redness [5]. The dynamic vascular reaction causes both the skin colour and thermal changes in the hand. Reaction to the immersion of hand in the ice cold water (2–4°C) is not only superficial but the anxiety feeling connected with lowering body temperature and emotional excitement are noticed along with the blood pressure and heart rate increase during the test [6, 7].

The visible skin reaction in the first and second phase is the ground for depicting the course of the Hines-Brown test by thermovisual body surface temperature assessment method. This diagnostic, noninvasive as well as more and more often used in medicine method is based on the imagery and analysis of infrared radiation emitted by the examined body surface [8, 9].

The aim of the work was a thermovisual assessment of the change of temperature on the surface of a hand during the Hines cold pressor test since no works dealing with the issue were found in the literature.

## 2. Material and Methods

Present study was carried out on 30 volunteer male students of Physiotherapy Department at the University School of Physical Education in Wrocław, in the age of 23.5 ± 0.8 years. The average weight of the examinees was 78.4 ± 9.2 kg, their height 180.7 ± 5.9 cms, and BMI 23.9 ± 2.2 kg/m². All the subjects have been the students of sports university and have undergone regular medical examinations and on the day of the test were healthy, well-rested and had refrained from alcohol or any other stimulants for at least two days prior to the examination and on the day of the test did not smoke cigarettes. None of the persons was treated due to cardiovascular, neurological, respiratory, or muscle disease. Prior permission number KB-1/2013 was taken from the Bioethical Commission at the Wroclaw Medical University.

The cold pressor test assessing the functional state of the autonomic nervous system was conducted in accordance with the Hines protocol. The test was performed in late October and early November in a closed room (gym) in the morning (10:00–12:00 AM). The air temperature was 20 degrees Celsius, relative humidity between 40 and 60%, and the room was ventilated naturally (through open windows). Blood pressure measurement was made prior to trial simultaneously with the execution of the thermal images. Then, the subject remained in the rest position (lying) on the medical examination couch for 10 minutes. Each subject was instructed to stay in silence in order to relax fully. Before dipping the right hand in water with a temperature no higher than 4° Celsius for one minute, a blood pressure and HR measurements were performed on a nontested left arm. Analogical tests were carried out 30 seconds after removing the hand from water as well as 60 seconds afterwards.

The subjects were made to undergo Hines-Brown cold pressor test, their systolic and diastolic blood pressure and heart rate were measured four times by a cuff device on the left arm. The measurements of blood pressure and heart rate done before and after the immersion were used to indicate the influence of the cold pressor test on the surface temperature of the hand. Each subject immersed their right, dominant hand in ice cold water. A thermovisual picture of both hands was taken with a thermovisual camera, ThermoVision A20M (FLIR System, USA, OR, Wilsonville) joined with a personal computer equipped with Therma CAM Researcher 2.8. software. Another thermovisual picture was taken in the 3–5 minute after finishing the test [5, 10]. The subjects did not report any feeling of pain and their blood results regained the output parameters in the second minute after the test.

Three researchers took part in the examinations, one of whom was responsible for blood pressure and HR measurement, the other for thermovisual pictures while the third dealt with time control and process monitoring.

The percentage of temperature change after cold pressor test was calculated with the usage of a formula where

(1) $$\begin{array}{c} R\%=\frac{T_{a}-T_{b}}{T_{b}}*100\%. \end{array}$$

*R*%—% of surface hand temperature change under the influence of CPT. *T*_*a*_—surface hand temperature after the CTP. *T*_*b*_—surface hand temperature before the CTP.

Statistica 10 software was used to compile the results. Descriptive statistics (average, SD) Wilcoxon test was applied to compare particular variables and *R*-Spearman correlation defining interdependence of anthropometric and blood parameters as well as hand surface temperature. The accepted significance level was *p* < 0.05.

## 3. Results

The blood pressure and heart rate were examined, in a lying position, before the hand immersion was on average: systolic 124.0 ± 7.7 mmHg, diastolic 73.1 ± 6.4 mmHg, and heart rate 67.3 ± 8.2 beats per minute. The blood parameters after the cold pressor test were higher and differed from the output in a statistically significant way. The biggest difference was registered at systolic blood pressure which raised 10.4 ± 4.9 mmHg on average. The results are shown in Table 1.

The average hand temperature before the CTP was 29.0°C and was the highest on the dorsal side of the left-untested hand (29.44 ± 1.31°C), but the lowest on the palmar side of the right hand (28.75 ± 1.58°C). Statistically significant differences in the change of surface temperature between the dorsal and palmar side of the left hand were observed (difference = 0.65°C, *p* < 0,001) as well as between the right and left hand on the dorsal side (difference = 0.41°C, *p* < 0,05).

After carrying out the cold pressor test, the temperature dropped both on the right-tested hand on the dorsal by 9°C whilst on the palmar side by 7.7°C on average, and on the left-untested hand by 0.78°C and 0.55°C, respectively.

The drops in temperature were not regular and differed in a statistically significant way regarding dorsal and palmar side of a hand. The mean values of differences between the drop in temperature on the dorsal and palmar side on the right hand were 1.26 ± 0.88°C (*p* < 0,001) whilst on the left 0.24 ± 0.32°C (*p* < 0,001). The changes of hand surface temperature after the cold pressor test as well as the statistical significance of the differences are shown in Table 2.

The percentage of the temperature change after CPT was calculated. The surface temperature after the Hines test decreased on the dorsal side by 31% and on the palmar side by 27% on average. Respectively, 2.57% and 2.0% drops in the untested hand were noticed.

A slight positive, statistically significant correlation between the age and heart rate difference before and after the CPT was found out (0.363943). There was no correlation, however, between the obtained blood parameters, mass and height of the body, BMI rate nor hand surface temperature.

Significant correlations between the systolic blood pressure and the decrease of temperature in the tested hand on the dorsal (0.530083) and palmar sides (0.579772) as well as diastolic blood pressure and the decrease of temperature in the untested hand on the palmar side (0.495889) were observed. The correlation between the blood pressure difference and temperature decrease is shown in Table 3.

No statistically significant correlation between the heart rate and the hand surface temperature drop of the tested hands was found. The correlation between the heart rate difference and temperature decrease is shown in Table 4.

High correlation was observed between the temperature drop (0.83623) and the percentage of the temperature change dependence (0.91306) of the right hand on the dorsal and palmar sides. The results are shown in Table 5.

## 4. Discussion

The concept of the Hines test is to determine the quantity of the blood pressure increase. Treble blood pressure measurement in the right conditions shows an initial increase of about 10–20 mmHg for about 3 minutes, which is followed by blood parameters normalization [11, 12]. These studies showed that there was a statistically significant increase in the systolic and diastolic blood pressure as well as the heart rate as a response to cooling which confirms previous research done during the Hines cold pressor test [1–4, 11].

It was shown that thanks to a simple, visible reaction to cold water, the cold pressor test is an effective predictor of cardiovascular dysfunctions. The noninvasive Hines-Brown's test can be used for the assessment of vascular reactivity in both healthy persons and patients with circulatory system diseases [4, 13].

The assessment of cardiovascular system reaction to the cold pressor test by using only blood pressure and heart rate measurement may be not enough to qualify patients for cryotherapy. The usage of thermovisual method allows to represent the surface skin reaction to an external temperature factor. Thermovisual method uses blood supply and consequently the temperature of the tissues subject to thermal stimulation as well as the disease process [14]. Due to that fact, thermovision is currently used in sports medicine, physiotherapy, occupational health or sport. The temperature of skin subject to a physiotherapy treatment (and to physical operation in particular) changes the most often during the treatment. The degree of temperature change depends on the distance and type of the treatment as well as on the vascularity of this body area [15–17].

Thermovision found a wide application in the analysis of the blood supply change connected with the physical effort of training sportsmen. It allows to plan training load properly in order to protect the body parts subject to injury [16].

Significant changes in the surface temperature of the tested hand were observed in the present study.

The comparison of thermovisual studies done on the patients subject to cryotherapy and to cold immersion test seems to be important. It is known both from our studies as well as the ones of other authors that average drops in body surface temperature in persons who have undergone whole-body cryostimulation in −140°C for 3 minutes accounted for about 20% in lower limbs, 5% in the trunk, and 10% in upper limbs [10, 18]. In case of CPT the changes oscillated between 25 and 30%. The difference of changes results from water and air physical qualities in which thermal stimulations are performed. Water environment, like in case of cold pressor test, has four times higher heat capacity than air (water heat conduction is 25 times higher than the one of air), in which cryostimulation is done. At the same time, the reaction to CTP is more rapid as cooling human body in water is 2-3 times faster than in air environment. Due to that also skin and vascular reaction is faster, more dynamic and the stimulation itself can be shortened to 1-2 minutes while producing similar effects to longer cryostimulation. The usage of water for thermal stimulation purposes demands, however, defining the maximum time of immersion as the heat loss to water in 250 times higher than to air [5].

Thermovisual body surface assessment method was used by many authors to evaluate effects of particular working conditions. Marszałek and Kowalski studied the changes of peripheral circulation in an arm subjected to vibrations [19]. Except for the reaction on a tested hand, they confirmed previous studies concerning thermal stimuli transmission from a hand undergoing external, physical factors to the untested hand [19, 20]. A similar thermal stimuli transmission was observed in the present study. A statistically significant surface temperature drop in the left-untested hand was noticed after the cold pressor test, which was dependent on the hand side.

While comparing the changes occurring on the dorsal and palmar side, significant differences between the temperature drops after the cold pressor test were observed. The lowest temperature was noticed on the dorsal side of a tested hand. This may result from the anatomical hand structure and in particular the amount of muscle tissue which appears on the palmar side. Statistically significant differences between dorsal and palmar sides in temperature drops were observed on both tested and untested hands. At the same time the tested hand temperature drops on the dorsal and palmar sides correlated significantly with the observed changes of systolic blood pressure. The statistically significant correlation between untested hand surface temperature drop and change of diastolic blood pressure, which was observed in the study, needs further investigation however. The correlation mentioned above may result from fear and stress reaction to body cooling.

## 5. Conclusion

1. Statistically significant increase of systolic and diastolic blood pressure and heart rate were observed in the Hines cold pressor test. The result confirms previous publications and reflects the sympathetic nervous tension associated with an increase in PRU.
2. Under the influence of cold pressor test a drop in hand surface temperature was observed in all subjects. It accounted for about 29% in the tested and 2% in the untested hand.
3. The drops in temperature were higher on the dorsal side and significantly differed from the drops on the palmar side which proves a great importance of the muscle tissue and its role in the prevention of cooling the body.

## Figures and Tables

**Table 1:** Blood pressure and heart rate before and after cold pressor test in the subjects.

*N* = 30		Mean ± SD	Rise	*p* value
SBP	Before	124.0 ± 7.7	10.4 ± 4.9	**p** < 0.0001
	After	134.4 ± 8.7		

DBP	Before	73.1 ± 6.4	7.9 ± 4.9	**p** < 0.0001
	After	81.0 ± 7.1		

HR	Before	67.3 ± 8.2	5.4 ± 2.2	**p** < 0.0001
	After	72.7 ± 8.3		

SBP = systolic blood pressure.

DBP = diastolic blood pressure.

HR = heart rate [beats/min].

**Table 2:** Average hand surface temperature before and after the cold pressor test.

*N* = 30			Mean ± SD	Difference ± SD	*p*	Difference ± SD	*p*
Right hand (tested)	Dorsal side	Before	29.03 ± 1.33	9.00 ± 1.64	**p** < 0.0001	1.26 ± 0.88	**p** < 0.0001
		After	20.03 ± 1.8				
	Palmar side	Before	28.75 ± 1.58	7.73 ± 1.62	**p** < 0.0001		
		After	21.01 ± 1.79				

Left hand (nontested)	Dorsal side	Before	29.44 ± 1.31	0.78 ± 0.25	**p** < 0.0001	0.24 ± 0.32	**p** < 0.001
		After	28.64 ± 1.25				
	Palmar side	Before	28.79 ± 1.45	0.55 ± 0.23	**p** < 0.0001		
		After	28.24 ± 1.43				

**Table 3:** The correlation between the variations of blood pressure. Heart rate and surface temperature difference before and after the cold pressor test.

*N* = 30		Systolic blood pressure	Systolic blood pressure	Heart rate
Right hand (tested)	Dorsal side	0.530083^**∗**^	0.011761	−0.147322
	Palmar side	0.579772^**∗**^	0.180637	−0.157786

Left hand (nontested)	Dorsal side	−0.133289	0.320823	−0.035817
	Palmar side	−0.051086	0.495889^**∗**^	0.217919

^*∗*^Statistically significant *p* < 0.05.

**Table 4:** The correlation between the heart rate and surface temperature difference before and after the cold pressor test.

		Heart rate
Right hand (tested)	Dorsal side	−0.147322
	Palmar side	−0.157786

Left hand (nontested)	Dorsal side	−0.035817
	Palmar side	0.217919

^*∗*^Statistically significant *p* < 0.05.

**Table 5:** The correlation between the hand surface temperature differences before and after the cold pressor test.

		Temperature decrease			Relative temperature change in percentage		
*n* = 30		Right hand	Left hand		Right hand	Left hand	
		Palmar side	Dorsal side	Palmar side	Palmar side	Dorsal side	Palmar side
Right hand (tested)	Dorsal side	0.83623^**∗**^	0.016915		0.91306^**∗**^		
	Palmar side			−0.004946	0.010029		

Left hand (nontested)	Dorsal side			0.080591		0.004460	0.166778

^*∗*^Statistically significant *p* < 0.05.
